# Life after Adolescent and Adult Moderate and Severe Traumatic Brain Injury: Self-Reported Executive, Emotional, and Behavioural Function 2–5 Years after Injury

**DOI:** 10.1155/2015/329241

**Published:** 2015-10-13

**Authors:** Torun Gangaune Finnanger, Alexander Olsen, Toril Skandsen, Stian Lydersen, Anne Vik, Kari Anne I. Evensen, Cathy Catroppa, Asta K. Håberg, Stein Andersson, Marit S. Indredavik

**Affiliations:** ^1^Regional Centre for Child and Youth Mental Health and Child Welfare-Central Norway, Faculty of Medicine, Norwegian University of Science and Technology (NTNU), 7491 Trondheim, Norway; ^2^Division of Mental Healthcare, Department of Child and Adolescent Psychiatry, St. Olavs Hospital, Trondheim University Hospital, 7006 Trondheim, Norway; ^3^Children's Clinic, St. Olavs Hospital, Trondheim University Hospital, 7006 Trondheim, Norway; ^4^MI Lab and Department of Circulation and Medical Imaging, Norwegian University of Science and Technology, 7491 Trondheim, Norway; ^5^Department of Physical Medicine and Rehabilitation, St. Olavs Hospital Trondheim University Hospital, 7006 Trondheim, Norway; ^6^Department of Neuroscience, Faculty of Medicine, Norwegian University of Science and Technology, 7491 Trondheim, Norway; ^7^Neuroclinic, Department of Neurosurgery, St. Olavs Hospital, Trondheim University Hospital, 7006 Trondheim, Norway; ^8^Department of Public Health and General Practice, Department of Laboratory Medicine, Children and Women's Health, Norwegian University of Science and Technology, 7491 Trondheim, Norway; ^9^Department of Physiotherapy, Trondheim Municipality, Norway; ^10^Child Neuropsychology, Murdoch Children's Research Institute, Melbourne, VIC 3052, Australia; ^11^Department of Pediatrics and School of Psychological Sciences, University of Melbourne, Melbourne, VIC 3010, Australia; ^12^Department of Psychosomatic Medicine, Oslo University Hospital, 0424 Oslo, Norway; ^13^Department of Psychology, University of Oslo, 0373 Oslo, Norway

## Abstract

Survivors of moderate-severe Traumatic Brain Injury (TBI) are at risk for long-term cognitive, emotional, and behavioural problems. This prospective cohort study investigated self-reported executive, emotional, and behavioural problems in the late chronic phase of moderate and severe TBI, if demographic characteristics (i.e., age, years of education), injury characteristics (Glasgow Coma Scale score, MRI findings such as traumatic axonal injury (TAI), or duration of posttraumatic amnesia), symptoms of depression, or neuropsychological variables in the first year after injury predicted long-term self-reported function. Self-reported executive, emotional, and behavioural functioning were assessed among individuals with moderate and severe TBI (*N* = 67, age range 15–65 years at time of injury) 2–5 years after TBI, compared to a healthy matched control group (*N* = 72). Results revealed significantly more attentional, emotional regulation, and psychological difficulties in the TBI group than controls. Demographic and early clinical variables were associated with poorer cognitive and emotional outcome. Fewer years of education and depressive symptoms predicted greater executive dysfunction. Younger age at injury predicted more aggressive and rule-breaking behaviour. TAI and depressive symptoms predicted Internalizing problems and greater executive dysfunction. In conclusion, age, education, TAI, and depression appear to elevate risk for poor long-term outcome, emphasising the need for long-term follow-up of patients presenting with risk factors.

## 1. Introduction

Adolescents and adults surviving moderate and severe Traumatic Brain Injury (TBI) often experience long-lasting cognitive, emotional, and behavioural problems [1–4]. In particular executive dysfunction has been demonstrated to have a profound impact on the ability to resume education, employment, and independent living [5–7]. Further, TBI is associated with an increased risk of developing symptoms of psychiatric disorders such as depression [8], anxiety [9], substance abuse [10], personality problems [3, 11], and behavioural changes such as aggression [12, 13]. All of those symptoms affect reintegration into the community [14], and therefore it is of great importance to identify those at risk for poorer long-term outcomes.

Executive function is a complex, overarching concept that refers to all functions related to goal-directed regulation of thoughts, actions, and emotions, including problem-solving, monitoring ongoing operations, switching between operations, emotion regulation, initiation of behaviour, and inhibition of nonadaptive behaviour [15, 16]. While some components of executive functions such as monitoring and switching cognitive operations may be assessed by standardized neuropsychological tests, other aspects such as regulating emotions and actions which are often more detrimental to adaptive functioning are often not captured by such tests [16, 17] and are better measured by questionnaires [18]. Further, the relationship between self-reported and performance-based executive function after TBI is far from established [19, 20].

Several self-report inventories have been developed aiming to assess not only the goal-directed regulation of thoughts, but also regulation of actions and emotions. Studies have demonstrated significant changes in self-reported executive function after TBI related to the individuals overall ability to regulate thoughts, emotions, and behaviour [21, 22]. However, there is a further need for studies utilizing more fine-tuned assessment tools providing more detailed profiles of typical executive problems after TBI. Although descriptions of typical profiles of self-reported executive problems have been examined as long as 10 years after childhood TBI [23, 24], there are no previous studies that have investigated such long-term consequences of TBI sustained in adolescence and adulthood.

Previous research has shown that a broad assessment is necessary to capture the variety of emotional and behavioural problems that persons may experience after TBI, including both Internalizing emotional problems such as depression or anxiety as well as Externalizing problems such as aggressive behaviour [1, 9, 13, 25]. In fact, compared with the general population, a substantially larger proportion of individuals with TBI qualify for an Axis I diagnosis according to DSM-IV, with depression, anxiety, and substance abuse most commonly observed [9, 18]. However, people with TBI may also experience a greater degree of subclinical symptoms compared to healthy individuals, which are better captured by questionnaires [18, 26]. Furthermore, while studies have described self-reported symptoms of depression [27], anxiety [28], and aggression [12] after TBI previously, there is a paucity of studies investigating Internalizing and externalizing simultaneously following TBI sustained in adolescence and adulthood.

Assessing cognitive, emotional, and behavioural changes after TBI using self-report gives access to unobservable, internal experiences to which only the person with TBI is privileged. A number of adult TBI studies have shown reasonable correspondence between self-report and family report [4, 29, 30]. However, it has been suggested that persons with severe injuries may underreport their problems which is hypothesised to be caused by reduced self-awareness or insight [31]. Nevertheless, the appropriateness of using proxy reports from family has been debated in the broad psychology literature, in particular for children and adolescents [32, 33], suggesting the possibility that family report reflects their own distress rather than that of the person with TBI [34, 35]. Further, it has been shown that the persons' own perception of their problems may influence how well they manage to reintegrate into the community [6, 36, 37]. Taken together, these findings warrant a focus on descriptions of typical self-reported problems as it could inform clinicians as to which symptoms should be targeted during rehabilitation and to inform general clinical decision making.

When considering possible predictors of self-reported executive problems after TBI sustained in late adolescence and adulthood, association has been found with length of education [38], lesion localization [29], Glasgow Coma Scale (GCS) score [39, 40], and concurrent emotional status (i.e., depression) [39]. However, to our knowledge reports of an association between long-term self-reported executive problems after a TBI sustained in adolescence and adulthood and duration of PTA and traumatic diffuse axonal injury (TAI) are lacking. Executive functions rely upon network interactions between several cortical, subcortical, and cerebellar brain regions [16, 41, 42], leaving them vulnerable to traumatic axonal injury (TAI) as a consequence of TBI [43–45]. Detecting TAI in vivo has been challenging, and it has further been demonstrated that valuable information about the magnitude of TAI may be lost if MRI is not performed in an early phase after injury [46]. While no relationship has been found between white matter integrity and concurrent self-reported executive function in the chronic phase after moderate and severe TBI utilizing diffusion tensor imaging (DTI) [47], better self-reported executive function in the chronic phase after moderate-to-severe TBI has been associated with compensatory brain activations as measured with functional magnetic resonance imaging (fMRI) [48]. However, no previous studies have investigated associations between self-reported executive function in the chronic phase and TAI as detected by clinical MRI in the early phase.

Associations with self-reported emotional and behavioural change after TBI are equally complex. Development of depression and anxiety after TBI has been observed to be associated with low socioeconomic resources (i.e., fewer years of education) [9, 25], while aggression and antisocial personality problems have been found to be associated with age [9, 26]. Evidence of associations between injury severity and later neuropsychiatric problems has been conflicting [9, 18, 49, 50], with some studies reporting no association at all [51]. Furthermore, the occurrence of mood disorders has been related to dysfunction in neural circuits involving cortical and subcortical structures [52], but only a few of those studies have included magnetic resonance imaging (MRI) findings [53, 54] and reviews in the field are inconclusive [9, 18].

This study adresses the gaps in the previous litterature by investigating long-term cognitive, emotional, and behavioural self-reported outcomes after moderate and severe TBI sustained in adolecense and adulthood by utilizing fine-tuned tools assessing a broad range of possible symtoms; providing methodologicaly sound methods such as prospective recruitment, comparisons to a large, matched control group; and providing high quality imaging methods for assessing the impact of TAI. The aims of this study were to (1) investigate long-term self-reported executive, emotional, and behavioural function after moderate-to-severe TBI sustained in late adolescence and adulthood and (2) to explore the association between demographic, injury-related, psychological, global outcome, and neuropsychological factors, as obtained in the postacute phase and later self-reported problems. We hypothesised that persons with TBI would report more overall problems with executive function as well as more symptoms of emotional and behavioural problems than healthy individuals 2–5 years after the injury. Based on previous literature using fine-tuned tools such as the Behaviour Rating Inventory of Executive Function-Adult version (BRIEF-A) [30, 38], we expected that problems with problem-solving [30] and working memory [38] would be among the most frequently reported executive cognitive problems. Moreover, we hypothesised that symptoms of depression, anxiety, and aggression would be frequently reported 2–5 years after injury. As the literature shows conflicting evidence of the impact of injury-related measures [39, 40, 47, 51, 53, 54], we specifically investigated the predictive value of injury severity measures such as GCS score, length of PTA, and TAI as detected by MRI in the early phase after TBI. While concurrent emotional status has been demonstrated to affect self-report [29, 55], we wished to extend previous findings by examining whether emotional status during the first year after injury could affect self-report as long as 2–5 years after injury. Based on previous findings [39, 40, 53], we hypothesised that measures of injury severity would be negatively associated with long-term self-reported executive, emotional, and behavioural function. Finally, we explored whether age at injury, length of education, performance-based cognitive function, global function as well as emotional status could explain some of the variance in outcome variables.

## 2. Methods

### 2.1. Study Design and Participants

From October 2004 to July 2008, 236 consecutive patients with moderate and severe TBI according to the Head Injury Severity Scale (HISS) criteria [56] were admitted to the Department of Neurosurgery at St. Olavs Hospital, Trondheim University Hospital, Norway, and registered in a database. Five did not consent to any follow-up. Participants registered in this database were contacted between February 2009 and August 2010 if they were more than one year after injury and fulfilled the inclusion criteria: (1) 15–65 years of age at the time of injury; (2) fluency in Norwegian; and (3) Glasgow Outcome Score Extended (GOSE) ≥5 at time of assessment (follow-up). Exclusion criteria were ongoing or preinjury substance abuse, neurological or psychiatric conditions, or previous moderate-to-severe TBI.

**Figure 1:**
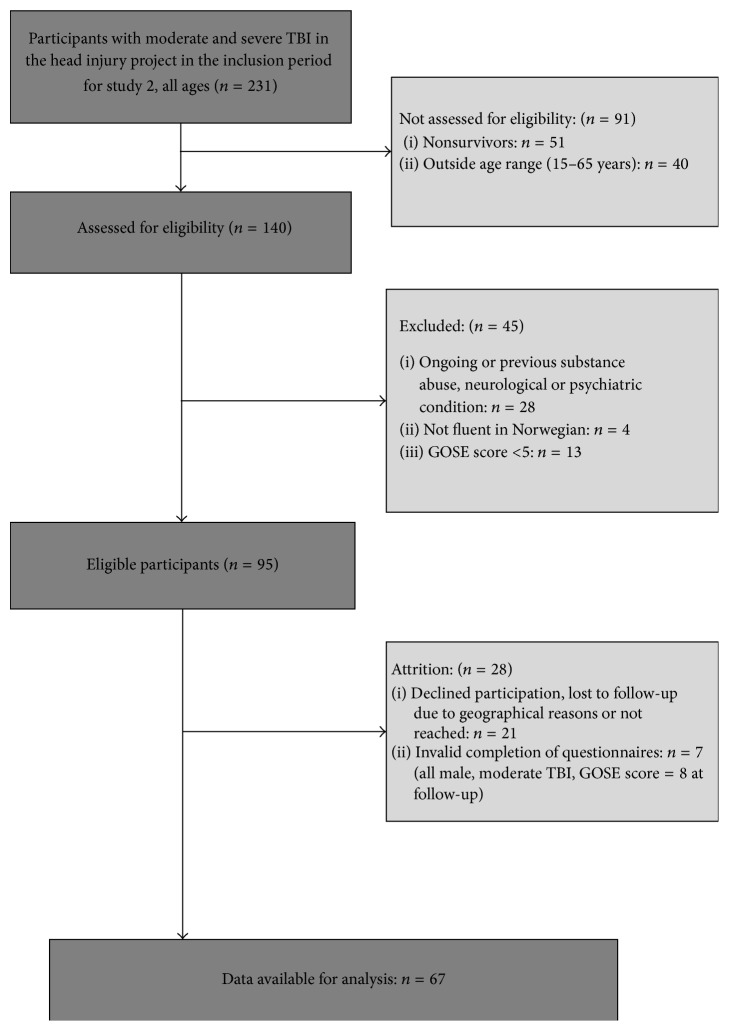
Flowchart illustrating sample selection and description of nonparticipants.

Of the 231 patients in the database, 51 died, and 40 were outside the age range. Forty-five were excluded because of premorbid or ongoing illness endorsed in the unstructured clinical interview during the hospital stay after the injury (*n* = 28), being not fluent in the Norwegian language (*n* = 4), and GOSE scores < 5 (*n* = 13). This left 95 patients eligible for this study, of which 74 (78%) consented to a single follow-up assessment between 2 and 5 years after injury. Seven were excluded from analysis owing to invalid questionnaire completion. This left 67 TBI survivors for the full analysis. There were no differences in the distribution of age, gender, education, or injury severity between participants and nonparticipants. Description of patient selection and nonparticipants as well as timeline is described in the flowchart in Figure 1.

Forty-nine patients (injured October 2004–October 2007) consented to participate in a study on longitudinal cognitive outcome with neuropsychological assessment and screening for depressive symptoms 3 and 12 months after injury, as well as participating in the follow-up study 2–5 years after injury. Eighteen participants (injured October 2004–October 2008) consented to participate in the 2–5 years after injury follow-up study, but not to participate in the assessments at 3 and 12 months after injury. There were no differences in the distribution of age, gender, education, or injury severity between the persons participating in the neuropsychological assessment + follow-up compared to those participating only in the follow-up, except that a larger proportion of participants in the first group had PTA durations of >1 week (Pearson's Chi-square, *p* = 0.042). There were no differences between the participants as a whole group compared to the nonparticipants.

Sex-, age-, and education-matched healthy control participants were recruited from the family and friends of the patients with TBI, hospital employees, and through advertisement. Six of 78 recruited controls were excluded because of previously diagnosed psychiatric or neurological conditions (discovered on the day of testing, *n* = 3) or invalid completion of the forms (*n* = 3). As a result, 72 control participants were included.

### 2.2. Material and Procedures

**Figure 2:**
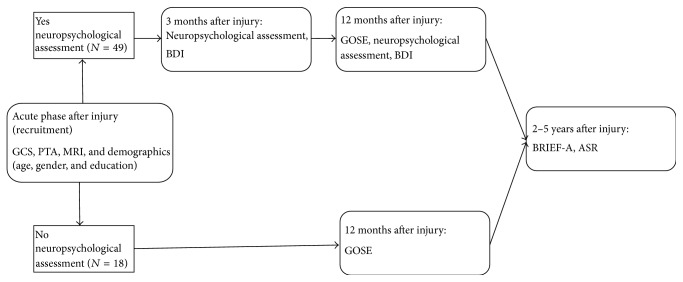
Timeline displaying the time points during data collection and the assessments for this study.

Figure 2 describes the timeline for the various measures.

#### 2.2.1. Long-Term Outcome Measures (2–5 Years after Injury)

Participants completed questionnaires that assessed self-reported executive, emotional, and behavioural problems at follow-up (mean 2.9 ± 0.9 years after injury, range: 2–5 years after injury). A few participants were unable to complete all questionnaires. While 17 (25%) of the participants were between 15 and 18 years of age at the time of injury (adolescents), all but one participant were ≥18 years of age when completing the questionnaires at follow-up 2–5 years after injury and one was 17 years old. We used a self-report form and an interview to estimate the number of years of education completed at the time of follow-up.

#### 2.2.2. Self-Reported Executive Function

Self-reported executive function was assessed with the BRIEF-A questionnaire, which consists of 75 items that measure behavioural, emotional, and cognitive aspects of executive function. It features sound psychometric properties [57, 58], good reliability, and large-scale norms [17, 58]. Each item is rated on a three-point frequency scale (0 = never; 1 = sometimes; 2 = often). Five items are designed to detect invalid response styles (inconsistencies or negativity). Seventy items generate three composite index scores and nine subscale scores. The subscales Inhibit, Shift, Emotional Control, and Self-Monitor generate the Behaviour Regulation Index (BRI), while the subscales Initiate, Working Memory, Plan/Organize, Task Monitor, and Organization of Materials constitute the Metacognitive Index (MI). In addition, a Global Executive Composite (GEC) is calculated from all 70 items. The BRIEF-A reference manual classifies the clinical range as *T*-score ≥65, with higher scores indicative of poorer function. The technical manual classifies a score on the negativity scale of >4 and a score on the inconsistency scale as >7 as an invalid report. Any reports that were classified as invalid according to these criteria were excluded from further analysis.

#### 2.2.3. Self-Reported Emotional and Behavioural Problems

Self-reported emotional and behavioural problems were assessed with the ASEBA: Adult Self-Report (ASR) Form [59]. The ASR consists of one section that measures adaptive functioning (38 items) and one section that measures emotional and behavioural problems (126 items) on a three-point scale (0 = statement not true; 1 = statement sometimes true; 2 = statement very true). Eight syndrome scales are generated: anxious/depressed, withdrawn, somatic complaints, thought problems, attention problems, aggressive behaviour, rule-breaking behaviour, and intrusive behaviour. The form yields three composite scores: Total problems, Internalizing problems (sum of the scales anxious/depressed, withdrawn, and somatic complaints), and Externalizing problems (sum of the scales aggressive, rule-breaking, and intrusive behaviour). The form also yields six DSM-IV-oriented scales: depressive, anxiety, somatic, avoidant personality, attention deficit hyperactivity disorder (ADHD), and antisocial personality problems. Items considered critical to diagnostic categories in the DSM-IV constitute the critical items scale. The ASEBA reference manual [59] recommends using raw scores when presenting descriptive data and borderline range using *T*-scores as the threshold in research (clinical cut-off) with higher scores indicative of poorer function. The clinical range is classified as *T*-score ≥ 70 and the borderline range is classified as *T*-score ≥ 65 for the syndrome scales; the respective ranges are classified as *T*-score ≥ 63 and ≥60 for the composite scales [59]. The subscales inattention and hyperactivity/impulsive are set at ≥97th percentile and ≥93rd percentile, respectively.

### 2.3. Measures of Predictors of Long-Term Outcome (at Injury and 12 Months after Injury)

#### 2.3.1. Injury-Related Variables: GCS Score, PTA, and Presence of TAI on Early MRI

GCS score was recorded at or after admittance if the patient deteriorated or before intubation in cases of prehospital intubation. GCS scores of 9–13 were classified as moderate TBI and scores ≤8 were considered severe TBI [56, 60]. Duration of PTA was categorized as ≤1 week or >1 week. The presence of TAI was assessed from the earliest MRI (1.5 Tesla) examination performed at median 10 days after injury (range = 1–120 days). The scan protocol included T1- and T2-weighted sequences, a T2^*∗*^-weighted gradient echo sequence, fluid-attenuated inversion recovery (FLAIR) sequences, and diffusion-weighted imaging. MRI parameters and the evaluation procedure have been reported in previous studies [61, 62].

#### 2.3.2. Global Function 12 Months after Injury

Global TBI related outcome was assessed with the Glasgow Outcome Scale Extended (GOSE) [63] structured interview at 12 months after injury for all participants recruited from the initial data base (*n* = 66).

#### 2.3.3. Subgroup Analyses: Neuropsychological and Emotional Assessment

The subgroup was assessed at 3 months after injury, with performance-based neuropsychological tests grouped into cognitive domains covering processing speed [64, 65], attention [66], memory [67–69], and executive function [65, 70].  Table 1 displays the cognitive domains and neuropsychological tests used. Raw scores were converted to *T*-scores applying normative data provided by the test manufacturers, except for the Symbol Digit Modality test in which a normative sample quoted by Lezak et al. [71] was used. Standardized scores on the individual neuropsychological tests were grouped into composite scores for each cognitive domain. *T*-scores were used in the analysis. These tests have demonstrated adequate validity and reliability [71]. The Vocabulary and Matrix Reasoning subtests of the Wechsler Abbreviated Scale of Intelligence (WASI) were used to estimate IQ [72]. Depressive symptoms were assessed with the Beck Depression Inventory (BDI) at both 3 months (*n* = 47) and 12 months (*n* = 44) after injury [73].

### 2.4. Ethics

The Regional Committee for Medical Research Ethics approved the study protocol. Written consent was obtained from patients aged ≥16 years at injury and from both participants and their parents if patients were aged <16 years at injury.

### 2.5. Statistical Methods

Demographic characteristics, injury severity characteristics, and the different cognitive domains are presented as mean (±standard deviation, SD) for normally distributed data, and otherwise as median with interquartile range (IQR; 25th to 75th percentile). For missing data, we used available case analysis, utilizing all cases for which the variables were present. We reported 95% confidence intervals (CIs) where relevant, and two-sided *p* values <0.05 were considered statistically significant. *p* values between 0.01 and 0.05 should be interpreted with caution owing to multiple hypotheses. Statistical analyses were performed with SPSS 18.0.

To describe differences in function between persons with TBI and controls, independent samples *t*-tests based on 2000 bootstrap samples were used. The Kruskal-Wallis test and Mann-Whitney *U* test were used for nonnormally distributed data. Effect sizes were calculated as Cohen's *d* based on pooled variance (*d*_pooled_) [74]. Cohen defined *d* of 0.8 as large, 0.5 as medium, and 0.2 as small effect sizes [75]. Differences in proportions were compared using the Chi-squared test, the unconditional *z*-pooled test [76], and the Newcombe confidence interval [77, 78].

To test associations between outcome measures and predictors, linear regression analyses were performed with composite scores from BRIEF-A and ASR as dependent variables; preinjury variables, injury-related variables, and GOSE scores were employed as covariates. In the subgroup analyses, neuropsychological test scores at 3 months after injury and BDI were employed as covariates. These covariates were included separately and then adjusted for age at injury and length of education at follow-up. An additional linear regression analysis was performed with main indexes and composite scores from BRIEF-A and ASR as dependent variables and the presence of TAI employed as a covariate with adjustment for BDI. Pearson's correlation coefficient (*r*) was used to analyse associations between the main indexes on BRIEF-A and the symptom scales on ASR.

## 3. Results

Participant characteristics are presented in Table 2 for the full sample and in Table 7 for the subsample. Individuals with TBI and healthy controls did not differ regarding distribution of sex, age at testing, or years of education. Participants with TBI assessed at 3 months after injury exhibited significantly lower estimated IQ and reduced processing speed, memory, and executive function compared with controls. At 2–5 years after injury a higher proportion of individuals with TBI neither worked nor attended school (18%) compared with controls (6%, difference in proportions: 12%; *p* = 0.03).

### 3.1. Self-Reported Executive Function 2–5 Years after Injury

Individuals with TBI reported more problems on all three composite indexes of BRIEF-A (GEC, BRI, and MI) than healthy controls (Table 3). Effect sizes were in the moderate range (0.38–0.66). More respondents with TBI (18%) reported symptoms in the clinical range on the GEC (difference in proportions; 17%, *p* < 0.001), BRI (8%; difference in proportions, 7%; *p* = 0.02), and MI (20%; difference in proportions, 18%; *p* < 0.001). On the BRI subscales, participants with TBI also reported more difficulties with inhibition, set-shifting, emotional regulation, and self-monitoring, with effect sizes in the medium range. On the MI subscales, individuals with TBI reported more problems with working memory than healthy controls, with 37% reporting working memory problems in the clinical range (difference in proportions, 32%; *p* < 0.001).

### 3.2. Emotional and Behavioural Outcome 2–5 Years after TBI

On the ASR adaptive scales, respondents with TBI reported significantly fewer personal strengths than healthy controls. They did not differ from controls with regard to problems in their family relationships or friendships. On the composite scales Total problems, Internalizing problems, and Externalizing problems, persons with TBI reported significantly more problems compared with controls (Table 4). Effect sizes were in the medium range (0.40–0.68). A greater proportion of individuals with TBI (20%) reported problems in the clinical range on the scales Total problems (difference in proportions, 18%; *p* = 0.002), Internalizing problems (24%; difference in proportions, 16%; *p* = 0.05), and Externalizing problems (14%; difference in proportions, 12%; *p* = 0.016). On the syndrome scales, individuals with TBI also reported more problems with anxiousness/depression, somatic complaints, thought problems, attention problems, and aggressive behaviour than healthy controls. Among the DSM-IV-oriented scales, respondents with TBI reported higher scores for depression, anxiety, somatic problems, and attention problems. They also reported higher scores than controls on critical items (*d*: 0.84).

### 3.3. Factors Associated with Executive, Emotional, and Behavioural Problems at Follow-Up

Fewer years of education predicted endorsement of greater problems on the GEC and BRI, but not on the MI (Table 5). TAI on MRI during the early phase predicted more problems on GEC and BRI, while GCS score and duration of PTA did not. However, the association between TAI and the GEC and BRI did not reach statistical significance when adjusted for age and education. Neuropsychological test performance at 3 months after injury was not associated with any of the BRIEF-A scales (*β* ranging from −0.187 to 0.137, *p* > 0.05 for all; see Table 8 for full overview). Depressive symptoms at 3 months after injury predicted metacognitive problems (MI) at follow-up, while depressive symptoms 1 year after injury predicted later executive problems on all the main indexes. Lower GOSE score at 12 months after injury predicted more problems on all main indexes at follow-up.

Younger age at injury predicted more emotional and behavioural problems at follow-up, particularly regarding Externalizing problems (Table 6). Presence of TAI on early MRI predicted higher scores on ASR Total problems and Internalizing problems. Only the association with Internalizing problems persisted after adjusting for age at injury and length of education. However, the presence of TAI still predicted higher scores on ASR Total problems and Internalizing problems, when adjusting for depressive symptoms 3 months after injury. More depressive symptoms at both 3 and 12 months after injury predicted later high scores on ASR Total problems, and depressive symptoms 12 months after injury predicted both Internalizing and Externalizing problems at follow-up.

Lower GOSE score at 12 months after injury predicted later high scores on both ASR Total problems and Internalizing problems when adjusted for age and education. Neuropsychological test performance at 3 months after injury was not associated with any of the ASR scales (*β* ranging from −0.086 to 0.588, *p* > 0.05 for all; see Table 8 for full overview).

Concurrent status of employment was not associated with any main BRIEF-A index or ASR composite score (*β*: −5.151 to 2.954, *p* > 0.05 for all). Patients that reported more problems on the ASR symptom scales also reported more problems on the GEC, with correlation coefficients ranging from 0.327 (thought problems) to 0.823 (attention problems; *p* < 0.001 for all). This pattern held true also for the indexes BRI (*r*: from 0.242 to 0.716, *p* < 0.01 for all) and MI (*r*: 0.283 to 0.816, *p* < 0.001 for all). An exception was ASR intrusive behaviour, which was associated only with BRI (*r*: 0.27, *p* = 0.027), and not with GEC (*r*: 0.20, *p* = 0.112) or MI (*r*: 0.12, *p* = 0.319; see Table 9 for full overview).

## 4. Discussion

In this large, prospective longitudinal study, our main aim was to delineate the magnitude and profile of chronic problems with executive, emotional, and behavioural function experienced by individuals with moderate-to-severe TBI 2–5 years after injury. As we hypothesised, greater overall self-reported executive problems were found among persons with TBI compared with healthy controls. This was evident both in terms of group differences and the frequency of individuals reporting problems in the clinical range. Further, persons with TBI significantly more often reported feeling sad or depressed compared with healthy controls. However, group differences in emotional and behavioural problems did not always indicate symptoms above the clinical cut-off, supporting emerging findings within the paediatric TBI population [79]. This observation suggests that subclinical executive problems are commonly experienced within the group as a whole, which may add to the total symptom burden for individuals with TBI.

Our study demonstrated that self-reported problems with working memory, attentional control, and monitoring ongoing operations were frequently reported among persons sustaining TBI in adolescence and adulthood, which adds to the findings in studies utilizing similar tools in populations with other neurological deficits [29, 80, 81]. We also observed that participants with TBI experienced significantly more problems with inhibition, mental flexibility, and emotional regulation, which adds to existing literature on adult/adolescent TBI populations applying the same assessment tools and has not been reported in previous studies. Contrary to our expectations, problem-solving, initiation, and task monitoring were not perceived as problematic among individuals with TBI in our study, which is in contrast to a study comprising moderate and severe TBI survivors, where these functions were perceived as most problematic [30]. However, the few studies that have employed the BRIEF-A as an outcome measure after TBI sustained in late adolescence and adulthood have had relatively small sample sizes [29, 30, 82], been retrospective in design [29, 83], and lacked comparisons with large demographically matched healthy control groups. We may speculate that by addressing these methodological issues our study revealed significant differences in self-reported problems within several areas of executive function after TBI previously not highlighted as problematic for this population.

While the presence of aggressive behaviour across the entire TBI group is in line with the literature reviewing long-term psychiatric outcome after TBI [13, 25], in our study, they did not report more rule-breaking behaviour (lack of empathy, substance abuse, and law-breaking behaviour) or intrusive behaviour. The aggression scale on ASR consists of several items related to behavioural control, and we speculate that executive problems (e.g., impaired inhibition and reduced task monitoring/switching) [15] may mediate the behavioural and emotional problems experienced by individuals after TBI [25]. Further, excessive mood swings were commonly reported, which may indicate an increased risk of psychiatric diagnoses [59]. Controlling emotional and behavioural expression is also important for social and occupational functioning [84]. However, our respondents with TBI did not report more social withdrawal or problems with social relations, which is in contrast to previous studies [9, 85]. It could be argued that the persons with TBI may have underestimated their social problems, but the substantial proportion of moderate TBI in our study may reduce the risk of underreporting problems due to problems with self-awareness [86]. Another possibility is that this measure may not be sensitive enough to pick up underlying relationship problems. However, the health care system in Norway provides early access to treatment and rehabilitation services, including family support interventions. Whether such access to early intervention may contribute to participants reporting less social problems compared to findings from previous studies should be investigated further in future research. Nevertheless, our finding illustrates that the persons with TBI were less concerned about relationship problems compared to problems with regulating their emotions and behaviour, which suggests that this area is an important target in post-TBI rehabilitation.

### 4.1. Factors Associated with Self-Reported Executive, Behavioural, and Emotional Problems

Our second aim was to explore the effects of demographic, injury-related, psychological, neuropsychological, and global outcome factors, as obtained in the postacute phase, on later self-reported problems. Firstly, we hypothesised that the measures of injury severity were associated with later long-term outcome. Extending previous studies, our results suggest that TAI plays a contributing role in the development of self-reported Internalizing problems (e.g., anxiety and depression) and behaviour regulation. This association persisted even after adjusting for early self-reported depressive symptoms. TAI is a microscopic strain injury of axons and blood vessels in different predilection locations of the brain, typically causing widespread damage often localized in frontotemporal and subcortical structures [87], also affecting subcortical structures with frontal projections [3, 88]. The same neuropathological changes may also affect the development of mood disorders [52]. In combination with the unexpected findings that other measures of injury severity were not associated with later self-reported problems [39], this suggests that the pathophysiological processes associated with TAI may have a distinct effect on later perceived problems with emotional and behavioural regulation as long as 2–5 years after injury. No previous study has investigated associations between long-term self-reported executive, emotional and behavioural function, and presence of TAI detected by clinical MRI in the early phase, and our findings suggest that neurological imaging in the early phase after TBI may aid in identifying persons at risk of poorer long-term outcome.

As hypothesised, we found that self-reported symptoms of depression within the first year after injury predicted later perceived overall problems with goal-directed cognitive and behavioural regulation, in addition to Externalizing and Internalizing problems. These findings support previous studies showing that emotional distress affects the extent of self-reported cognitive problems [29, 55]. However, adaptive problems in every-day life due to impairments after TBI in combination with the negative thinking typically experienced during depression [89] may contribute to the long-term self-reported depressive symptoms found in our study. This illustrates the importance of identifying, monitoring, and possibly treating depressive symptoms early in the course after TBI.

Further, younger age at injury predicted more self-reported Externalizing problems in the longer-term post-TBI, which is in accordance with previous studies that employed methods of retrospective assessment [12] or cluster analysis [26]. While we found that fewer years of education were associated with more self-reported problems with goal-directed cognitive and behavioural regulation, no association was found with self-reported aggressive behaviour. This is in contrast to other reports which have indicated that more years of education and higher socioeconomic status are associated with lesser endorsement of behavioural problems [26]. The aggressive behaviour in participants who were younger at the time of injury could be explained by the increased vulnerability to injury in rapidly developing brain areas [90]. The frontal lobe is still maturing during adolescence and young adulthood, rendering functions located therein (e.g., emotional and behavioural regulation) at increased risk following injury [91]. Furthermore, age was not associated with symptoms of depression, anxiety, or somatic complaints among individuals with TBI. Some have suggested the presence of distinct pathways and risk factors in the development of depression and anxiety as opposed to aggression [26], and we speculate that our findings might be in line with this. Our results indicate a need for future research to examine the possible differences in long-term outcome for persons injured in adolescence compared to those sustaining a TBI in adulthood.

We hypothesised that both global function and neuropsychological performance within the first year after injury would be associated with outcome 2–5 years after TBI. As expected, reduced global outcome one year after injury was associated with more reported executive and Internalizing problems. Experiencing reduced global function, including less ability to resume social relationships or leisure activities, may lead to a negative self-image and the increased endorsement of problems. However, the reported executive problems may also reflect cognitive impairment caused by the injury. Contrary to what we hypothesised, we observed no association between performance-based measures of cognitive function three months after injury and later self-reported executive, emotional, and behavioural function. This is in contrast to other studies demonstrating associations between performance-based and self-reported measures of task monitoring and switching [29, 30]. The lack of convergence among the data may be explained by different modes of measurement [92]. Our findings support the notion that self-reported cognitive complaints are affected by emotional symptoms [21, 29, 93], compared to performance-based measures of executive function which as previous studies suggest are more closely linked to neural damage after TBI [21, 29, 47]. Taken together, this may suggest that performance-based executive function may better reflect the efficacy of processing (optimal performance) as supported by the underlying brain structure [19], whereas self-reported executive function is rather related to adaptive functional changes in the brain [47, 48], possibly developing over time as a consequence of the initial injury and/or cognitive problems. Given the multifaceted and complex nature of executive dysfunction after TBI, further validation of both performance-based and self-report measures of executive function is needed.

### 4.2. Clinical Implications

By assessing self-reported executive, emotional, and behavioural long-term outcome, our study revealed that the persons with TBI experienced subjective problems that were not detected with, for instance, neuropsychological tests, illustrating the importance of including self-evaluation inventories in addition to tests. Our results indicate that detection of DAI on early MRI and assessment of self-reported symptoms of depression within the first year after injury can aid in identifying persons at risk of experiencing poorer executive, emotional, and behavioural long-term outcome. This can be used for more targeted and cost efficient rehabilitation. Furthermore, psychological and/or pharmaceutical interventions, with a focus on depressive symptomatology, may be helpful in reducing the long-term problems experienced by persons sustaining a TBI and lessen their overall symptom burden. In addition, the results suggest that age is a notable risk factor for development of aggressive behaviour, and initiating interventions targeting this should be part of the rehabilitation of adolescence sustaining TBI.

### 4.3. Study Limitations

Our main aim was to explore long-term change in self-reported executive, emotional, and behavioural function after moderate and severe TBI sustained in adolescence as well as in adulthood, which also guided the development of the study design. The reliance on self-report forms may limit transferral to studies applying performance-based neuropsychological tests or diagnostic interviews and makes our study less optimal for exploring the validity of the BRIEF-A and ASR as proxies for cognitive function and psychiatric diagnoses. Performance-based measures were available for only the subgroup that consented to participate in both the neuropsychological study and the long-term follow-up study. Due to this design, the findings should be interpreted with caution.

## 5. Conclusion

Persons with moderate and severe TBI reported significantly more pronounced difficulties in aspects of executive functions related to attentional control, working memory, and emotional regulation, as well as emotional and behavioural problems related to symptoms of depression, anxiety, and aggressive behaviour 2–5 years after injury compared to healthy controls. Both the presence of TAI on early MRI and reported symptoms of depression during the first year after injury were important predictors of later self-reported executive, emotional, and behavioural problems. Our findings indicate that demographic, neuropathological, and psychological factors all influence the development of self-reported executive, emotional, and behavioural problems for years after TBI. Our study highlights that early radiological and broad psychological evaluations may give clues as to which patients may be at risk for poorer long-term outcome. In summary, this study yields new information to guide the clinical management of TBI survivors and provides groundwork for additional clinical research regarding the long-term consequences of TBI.

## Figures and Tables

**Table 1:** Overview of performance-based neuropsychological tests assessing cognitive function in the subsample grouped into cognitive domains 3 months after TBI.

Neuropsychological tests			Reference
*Motor function*			
Grooved Pegboard	Dominant hand		[63]
*Information processing speed*			
Delis Kaplan Executive Function System		(D-KEFS)	[65]
Trail Making Test	Condition 2 (number sequencing) Condition 3 (letter sequencing)	(TMT)	
Color-Word Interference Test	Condition 1 (color naming) Condition 2 (word reading)	(CWIT)	
Symbol Digit Modality Test	Oral version Written version	(SDMT)	[64]
*Attention*			
Conners' Continuous Performance Test II		(CPT-II)	[66]
*Visual memory*			
Continuous Visual Memory Test		(CVMT)	[69]
Rey-Osterrieth Complex Figure Test		(ROCF)	[68]
*Verbal memory*			
California Verbal Learning Test-II		(CVLT-II)	[67]
*Executive function*			
Wisconsin Card Sorting Test computer version		(WCST)	[70]
Verbal Fluency Test (D-KEFS)	Condition 1 (letter fluency), Condition 3 (category change)		[65]
TMT (D-KEFS)	Condition 4 (number-letter sequencing)		
CWIT (D-KEFS)	Condition 3 (inhibition) Condition 4 (inhibition/switching)		
Tower Test (D-KEFS)			

**Table 2:** Description of participants: demographics, injury severity characteristics, and clinical observations at 1 and 2–5 years after moderate and severe TBI: global outcome and employment.

Variable	*n*	Persons with TBI	*n*	Controls	*p* value
*Demographics at injury*					
Male sex (*n*, %)	67	48 (72)	72	55 (76)	0.593^*∗*^
Age (mean, range)	67	29 (15–63)			
Injury-related variables					
Mechanisms of injury	67				
Traffic accident (*n*, %)		33 (49)			
Fall (*n*, %)		27 (40)			
Ski accident (*n*, %)		2 (3)			
Other (*n*, %)		5 (9)			
GCS score (median, IQR)	67	9 (7)			
HISS grade; moderate TBI (*n*, %)	67	39 (58)			
PTA <1 week (*n*, %)	66	37 (55)			
Early MRI findings	65				
EDH only (*n*, %)		1 (2)			
Pure TAI (*n*, %)		17 (25)			
Cortical contusions (*n*, %)		16 (24)			
Cortical contusions/TAI (*n*, %)		30 (45)			
Global outcome 12 months after injury					
GOSE score (median, IQR)	66	7.0 (2)			
*Demographics at follow-up*					
Age (mean, range)	67	32 (17–65)	72	33 (13)	0.683^†^
Years after injury (mean, SD)	67	2.9 0.8			
Years education (mean, range)	67	12 (9–18)	72	12 (2)	0.979^†^
Occupation	67		72		0.025^‡^
Unemployed/no school (*n*, %)		12 (18)		4 (6)	
Employed or at school (*n*, %)		55 (82)		68 (94)	

GCS: Glasgow Coma Scale; GOSE: Glasgow Outcome Scale Extended; IQR: interquartile range; PTA: posttraumatic amnesia; SD: standard deviation; TAI: traumatic axonal injury; TBI: Traumatic Brain Injury.

^*∗*^Pearson's Chi-squared test.

^†^Independent samples *t*-test.

^‡^Unconditional *z*-pooled test.

**Table 3:** Self-reported executive function on BRIEF-A at 2–5 years after moderate and severe Traumatic Brain Injury compared to healthy controls^*∗*^.

BRIEF-A (*T*-scores)	Persons with TBI		Controls		Mann-Whitney test	Effect size
	*n* = 67		*n* = 72			
	Mean	SD	Mean	SD	*p* value	*d* ^†^
Global scales						
Global Executive Composite (GEC)	51.40	(11.94)	46.19	(7.28)	**0.003**	0.53
Behaviour regulation Index (BRI)	50.69	(11.13)	44.51	(7.28)	**<0.001**	0.66
Metacognitive Index (MI)	51.81	(11.90)	48.02	(7.57)	**0.029**	0.38
Behavioural and emotional regulation scales						
Inhibit	51.84	(10.57)	47.72	(8.87)	**0.014**	0.42
Shift	49.52	(11.04)	44.64	(7.01)	**0.003**	0.53
Emotional regulation	51.61	(11.45)	44.88	(7.81)	**<0.001**	0.69
Self-Monitor	47.87	(10.70)	44.54	(7.74)	**0.039**	0.36
Metacognitive Index Scales						
Initiate	51.87	(11.68)	48.61	(9.86)	0.079	0.30
Working Memory	57.48	(13.01)	47.89	(7.91)	**<0.001**	0.89
Plan/Organize	50.54	(11.00)	47.61	(7.45)	0.071	0.31
Task Monitoring	50.97	(11.90)	48.88	(7.27)	0.217	0.21
Organization of Materials	46.60	(11.54)	48.49	(8.35)	0.268	−0.19

Higher *T*-scores indicate more problems.

^*∗*^Central tendency and variance given as mean and SD.

^†^Cohen's *d*.

SD: standard deviation.

**Table 4:** Self-reported adaptive function, personal strengths, and psychological problems on ASR at 2–5 years after moderate and severe Traumatic Brain Injury compared to healthy controls.

Adult Self-Report	Persons with TBI		Controls		Mean difference		*t*-test	Effect size
	*n* = 66		*n* = 71		(95 % CI)^†^			
	Mean	SD	Mean	SD	Lower	Upper	*p*	*d* ^‡^
Adaptive scores^*∗*^								
Personal strengths	16.18	(3.18)	17.39	(3.09)	−2.26	−0.15	**0.025**	**0.39**
Mean adaptive	49.61	(5.44)	50.15	(4.32)	−2.21	1.13	0.523	0.11
Relation to friends	9.82	(2.00)	10.00	(1.82)	−0.82	0.46	0.580	0.09
Relation to family	1.57	(0.44)	1.49	(0.37)	−0.06	0.22	0.246	−0.20
Composite scales								
Total problems	39.17	(26.08)	26.13	(16.67)	5.57	20.51	**0.001**	**0.60**
Internalizing problems	12.44	(9.81)	7.08	(5.42)	2.64	8.07	**<0.001**	**0.68**
Externalizing problems	9.05	(8.59)	6.24	(5.05)	0.40	5.22	**0.023**	**0.40**
Critical items	4.95	(3.64)	2.46	(2.22)	1.46	3.52	**<0.001**	**0.83**
Syndrome scales								
Anxious/depressed	6.48	(6.29)	3.34	(3.26)	1.43	4.87	**<0.001**	**0.63**
Withdrawn	2.27	(2.22)	1.75	(1.93)	−0.17	1.27	0.139	.25
Somatic complaints	3.68	(2.81)	2.00	(2.08)	0.84	2.52	**<0.001**	**0.68**
Thought problems	2.09	(2.26)	1.01	(2.25)	0.31	1.84	**0.006**	**0.48**
Attention problems	7.73	(5.37)	4.96	(3.72)	1.19	4.32	**0.001**	**0.60**
Aggressive behaviour	4.76	(4.55)	2.01	(2.46)	1.49	4.00	**<0.001**	**0.75**
Rule-breaking behaviour	2.70	(3.49)	2.21	(2.12)	−0.50	1.47	0.331	0.17
Intrusive behaviour	1.59	(1.96)	2.01	(1.89)	−1.07	0.23	0.200	−0.22
DSM-IV oriented scales								
Depression	5.02	(4.52)	2.70	(2.47)	1.06	3.56	**<0.001**	**0.64**
Anxiety	3.12	(2.67)	2.00	(2.00)	0.32	1.92	**0.007**	**0.47**
Somatic	2.21	(2.17)	1.30	(1.57)	0.28	1.55	**0.005**	**0.48**
Avoidant personality problems	2.32	(2.02)	2.11	(1.88)	−0.45	0.86	0.538	0.11
ADHD problems	7.20	(4.86)	4.85	(3.69)	0.88	3.82	**0.002**	**0.54**
Inattention	3.88	(2.81)	2.37	(2.10)	0.67	2.36	**0.001**	**0.61**
Hyperactivity/impulsivity	3.32	(2.53)	2.48	(2.21)	0.04	1.64	**0.040**	**0.35**
Antisocial personality problems	3.50	(4.44)	2.66	(2.73)	−0.41	2.11	0.182	0.23

Central tendency and variance measured in mean and SD (raw scores). Higher scores indicate more problems.

^*∗*^Higher scores indicate better function

^†^Results from *t*-test based on 2000 bootstrap samples.

^‡^Cohen's *d*.

ADHD: attention deficit and hyperactivity disorder, CI: confidence interval, DSM-IV: Diagnostic and Statistical Manual of Mental Disorders, 4th edition, and SD: standard deviation.

**Table 5:** Demographic and clinical factors during 1st year after injury associated with self-reported executive function (BRIEF-A) 2–5 years after moderate and severe TBI.

		Dependent variable							
		Regression coefficient for worse outcome, unadjusted				Regression coefficient for worse outcome, adjusted^*∗*^			

	Independent variables								
	*N*	*R* ^2^	Estimate	95% CI	*p* value	*R* ^2^	Estimate	95% CI	*p* value

Global Executive Composite									
Age at injury	67	0.049	−0.405	−0.850 to 0.040	0.074				
Years of education at injury	67	0.067	−3.076	−5.934 to −0.219	**0.035**				
PTA duration (1 week)	66	0.000	−1.103	−11.832 to 14.038	0.856	0.114	−1.273	−14.425 to 11.880	0.847
GCS score	67	0.003	−0.421	−2.279 to 1.437	0.652	0.098	−0.011	−1.861 to 1.838	0.990
Presence of TAI	65	0.063	14.140	0.279 to 28.002	**0.046**	0.150	10.616	−3.164 to 24.396	0.129
Presence of TAI adjusted for BDI 3 months after injury	48	0.048	12.012	−2.957 to 26.980	0.113				
BDI 3 months after injury	48	0.066	1.442	−0.192 to 3.075	0.082	0.117	1.579	−0.010 to 3.167	0.051
BDI 1 year after injury	45	0.277	2.224	1.105 to 3.343	**<0.001**	0.337	2.070	0.941 to 3.199	**0.001**
GOSE score 1 year after injury	67	0.087	−6.720	−12.161 to −1.279	**0.016**	0.231	−9.280	−14.945 to −3.615	**0.002**
Behaviour Regulation Index									
Age at injury	67	0.040	−0.159	−0.352 to 0.033	0.103				
Years of education	67	0.063	−1.277	−2.496 to −0.059	**0.040**				
PTA duration (1 week)	66	0.003	−1.148	−6.691 to 4.396	0.680	0.109	−1.806	−7.351 to 3.738	0.517
GCS score	67	0.005	−0.229	−1.030 to 0.572	0.570	0.089	−0.069	−0.870 to 0.733	0.864
Presence of TAI	65	0.059	6.677	0.721 to 12.633	**0.029**	0.143	5.313	−0.648 to 11.275	0.080
Presence of TAI adjusted for BDI 3 months after injury	49	0.129	6.314	0.286 to 12.343	**0.040**				
BDI 3 months after injury	49	0.043	0.478	−0.195 to 1.152	0.159	0.160	0.546	−0.108 to 1.200	0.099
BDI 1 year after injury	45	0.326	1.046	0.583 to 1.509	**<0.001**	0.381	0.979	0.513 to 1.443	**<0.001**
GOSE score 1 year after injury	67	0.087	−2.910	−5.241 to −0.578	**0.015**	0.216	−3.954	−6.417 to −1.490	**0.002**
Metacognitive Index									
Age at injury	67	0.048	−0.247	−0.520 to 0.026	0.075				
Years of education	67	0.053	−1.665	−3.410 to 0.080	0.061				
PTA duration (1 week)	66	0.000	0.242	−7.670 to 8.1559	0.951	0.099	−0.796	−8.740 to 7.148	0.842
GCS score	67	0.002	−0.192	−1.335 to 0.950	0.737	0.086	0.063	−1.080 to 1.206	0.913
Presence of TAI	65	0.047	7.501	−1.036 to 16.038	0.084	0.130	5.475	−2.964 to 13.961	0.202
Presence of TAI adjusted for BDI 3 months after injury	49	0.107	5.825	−3.438 to 15.088	0.212				
BDI 3 months after injury	49	0.055	0.968	−0.037 to 1.972	0.059	0.169	1.027	0.041 to 2.014	**0.042**
BDI 1 year after injury	45	0.200	1.136	0.437 to 1.834	**0.002**	0.248	1.029	0.318 to 1.741	**0.006**
GOSE score 1 year after injury	67	0.068	−3.653	−7.009 to −0.298	**0.033**	0.197	−5.244	−8.796 to −1.692	**0.004**

^*∗*^Adjusted for age at injury and years of education prior to the injury.

BDI: Beck Depression Inventory; BRIEF-A: Behavioural Rating Inventory for Executive Function-Adult version; CI: confidence interval; GCS: Glasgow Coma Scale; GOSE: Glasgow Outcome Scale Extended; PTA: posttraumatic amnesia; TAI: traumatic axonal injury; TBI: Traumatic Brain Injury.

**Table 6:** Demographic and clinical factors during 1st year after injury associated with self-reported emotional and behavioural problems (ASR) 2–5 years after moderate and severe Traumatic Brain Injury (TBI).

		Dependent variable							
		Regression coefficient for worse outcome, unadjusted				Regression coefficient for worse outcome, adjusted^*∗*^			

	Independent variables								
	*N*	*R* ^2^	Estimate	95% CI	*p* value	*R* ^2^	Estimate	95% CI	*p* value

ASR Total problems									
Age at injury	66	0.116	−0.640	−1.081 to −0.199	**0.005**				
Years of education at injury	66	0.032	−2.157	−5.143 to 0.828	0.154				
PTA duration (1 week)	64	0.000	−0.796	−14.013 to 12.420	0.905	0.155	4.782	−8.259 to 17.823	0.466
GCS score	66	0.000	0.085	−1.823 to 1.993	0.929	0.141	0.761	−1.090 to 2.613	0.414
Presence of TAI	64	0.076	16.085	1.822 to 30.347	**0.028**	0.185	12.728	−1.276 to 26.733	0.074
Presence of TAI adjusted for BDI 3 months after injury	46	0.315	15.524	1.772 to 29.277	**0.028**				
BDI 3 months after injury	46	0.235	2.845	1.303 to 4.388	**0.001**	0.331	2.868	1.375 to 4.362	**<0.001**
BDI 1 year after injury	43	0.401	2.683	1.662 to 3.705	**<0.001**	0.440	2.518	1.481 to 3.554	**<0.001**
GOSE score 1 year after injury	66	0.020	−3.308	−9.087 to 2.471	0.257	0.210	−7.371	−13.303 to −1.440	**0.016**
ASR Internalizing problems									
Age at injury	66	0.023	−0.106	−0.281 to 0.068	0.228				
Years of education	66	0.006	−0.343	−1.481 to 0.795	0.550				
PTA duration (1 week)	64	0.001	0.495	−4.416 to 5.405	0.841	0.041	1.380	−3.783 to 6.544	0.595
GCS score	66	0.000	0.006	−0.712 to 0.724	0.986	0.027	0.118	−0.623 to 0.859	0.752
Presence of TAI	64	0.075	5.986	0.668 to 11.303	**0.028**	0.097	5.548	0.055 to 11.041	**0.048**
Presence of TAI adjusted for BDI 3 months after injury	46	0.200	6.714	0.641 to 12.787	**0.031**				
BDI 3 months after injury	46	0.110	0.794	0.114 to 1.474	**0.023**	0.137	0.803	0.110 to 1.496	**0.024**
BDI 1 year after injury	43	0.306	0.922	0.490 to 1.354	**<0.001**	0.316	0.886	0.436 to 1.337	**<0.001**
GOSE score 1 year after injury	66	0.040	−1.764	−3.916 to 0.387	0.106	0.104	−2.768	−5.145 to −0.392	**0.023**
ASR Externalizing problems									
Age at injury	66	0.128	−0.221	−0.365 to −0.077	**0.003**				
Years of education	66	0.042	−0.819	−1.797 to 0.159	0.099				
PTA duration (1 week)	64	0.000	0.001	−4.373 to 4.375	1.000	0.174	2.029	−2.239 to 6.297	0.345
GCS score	66	0.001	−0.058	−0.686 to 0.570	0.853	0.157	0.171	−0.434 to 0.776	0.574
Presence of TAI	64	0.023	2.915	−1.859 to 7.689	0.227	0.146	1.630	−3.037 to 6.297	0.488
Presence of TAI adjusted for BDI 3 months after injury	46	0.298	3.525	0.485 to 1.414	0.106				
BDI 3 months after injury	46	0.255	0.917	0.446 to 1.388	**<0.001**	0.369	0.934	0.485 to 1.383	**<0.001**
BDI 1 year after injury	43	0.403	0.761	0.472 to 1.049	**<0.001**	0.446	0.718	0.426 to 1.010	**<0.001**
GOSE score 1 year after injury	66	0.002	−0.307	−2.228 to 1.613	0.750	0.178	−1.450	−3.442 to 0.542	0.151

^*∗*^Adjusted for age at injury and years of education prior to the injury.

ASR: Adult Self-Report (ASEBA); BDI: Beck Depression Inventory; CI: confidence interval; GCS: Glasgow Coma Scale; GOSE: Glasgow Outcome Scale Extended; PTA: posttraumatic amnesia; TAI: traumatic axonal injury; TBI: Traumatic Brain Injury.

**Table 7:** Description of participants in the subgroup analysis: demographics, injury severity characteristics, and clinical observations at 3 months, 1 year, and 2–5 years after moderate and severe TBI: cognitive function, emotional function, global outcome, and employment.

Variable	*n*	Persons with TBI	*n*	Controls	*p* value
*Demographics*					
Male sex (*n*, %)	49	35 (71)	28	24 (86)	0.593^*∗*^
Age at injury (mean, range)	49	30 (14–63)			
Injury-related variables					
GCS score (median, IQR)	49	9 (6)			
HISS grade; moderate TBI (*n*, %)	49	28 (57)			
PTA <1 week (*n*, %)	48	23 (47)			
Early MRI findings	48				
EDH only (*n*, %)		1 (2)			
Pure TAI (*n*, %)		10 (20)			
Cortical contusions (*n*, %)		14 (29)			
Cortical contusions/TAI (*n*, %)		23 (48)			
Neuropsychological assessment (3 months after injury)					
Days after injury (mean, SD)	49	99 (10)			
Estimated IQ (mean, SD)	47	106 (16)	26	119 (12)	0.001^†^
Processing speed (mean, SD)	46	44.5 (10.2)	26	53.0 (4.8)	<0.001^†^
Attention (mean, SD)	46	49.9 (4.9)	26	51.6 (4.3)	0.124^†^
Memory (mean, SD)	46	42.6 (10.0)	26	48.2 (8.3)	0.016^†^
Executive function (mean, SD)	47	47.3 (7.6)	26	53.1 (4.8)	0.001^†^
Depressive symptoms and global outcome 1st year after injury					
BDI 3 months after injury (mean, SD)	47	5.5 (4.4)			
BDI 12 months after injury (mean, SD)	44	6.7 (6.4)			
GOSE score 12 months after injury (median, IQR)	49	7.0 (2)			
*Demographics at follow-up*					
Years after injury (mean, SD)	49	3.2 1.0			
Age (mean, range)	49	34 (17–65)	28	34 (19–64)	0.895^†^
Years education (mean, range)	49	12 (9–18)	28	12 (9–18)	0.630^†^
Occupation	49		27		
Unemployed/no school (*n*, %)		10 (20)		1 (4)	
Employed or at school (*n*, %)		55 (82)		26 (96)	

GCS: Glasgow Coma Scale; GOSE: Glasgow Outcome Scale Extended; IQ: Intelligence Quotient; IQR: interquartile range; PTA: posttraumatic amnesia; SD: standard deviation; TAI: traumatic axonal injury; TBI: Traumatic Brain Injury.

^*∗*^Pearson's Chi-squared test.

^†^Independent samples *t*-test.

**Table 8:** Associations between main composite scores on BRIEF-A and ASR 2–5 years after moderate and severe TBI and neuropsychological test performance 3 months after injury^*∗*^.

			Dependent variable		
			Regression coefficient for worse outcome		

	Independent variable				
	*N*	*R* ^2^	Estimate	95% confidence interval	*p* value

BRIEF-A GEC					
Processing speed	47	0.015	−0.143	−0.492 to 0.205	0.413
Attention	47	0.001	−0.077	−0.831 to 0.678	0.839
Memory	46	0.008	0.112	−0.268 to 0.491	0.556
Executive function	48	0.012	−0.167	−0.626 to 0.291	0.467
BRIEF-A BRI					
Processing speed	47	0.015	−0.138	−0.470 to 0.194	0.408
Attention	47	0.006	−0.187	−0.887 to 0.513	0.594
Memory	46	0.004	0.074	−0.285 to 0.433	0.679
Executive function	48	0.011	−0.155	−0.592 to 0.281	0.478
BRIEF-A MI					
Processing speed	47	0.010	−0.115	−0.454 to 0.224	0.497
Attention	47	0.000	0.011	−0.723 to 0.745	0.976
Memory	46	0.013	0.137	−0.225 to 0.500	0.449
Executive function	48	0.009	−0.146	−0.591 to 0.299	0.513
ASR Total problems					
Processing speed	46	0.000	0.059	−0.753 to 0.872	0.883
Attention	46	0.000	0.065	−1.627 to 1.756	0.939
Memory	46	0.046	0.588	−0.229 to 1.405	0.154
Executive function	47	0.002	0.145	−0.933 to 1.223	0.788
ASR Internalizing problems					
Processing speed	46	0.010	0.106	−0.209 to 0.421	0.501
Attention	46	0.000	0.043	−0.620 to 0.705	0.897
Memory	46	0.060	0.260	−0.054 to 0.575	0.102
Executive function	47	0.008	0.126	−0.291 to 0.543	0.545
ASR Externalizing problems					
Processing speed	46	0.001	−0.033	−0.291 to 0.225	0.798
Attention	46	0.002	−0.086	−0.628 to 0.456	0.750
Memory	46	0.020	0.125	−0.138 to 0.388	0.344
Executive function	47	0.000	0.024	−0.318 to 0.367	0.887

^*∗*^Given in *T*-scores.

BRI: Behaviour Regulation Index, GEC: Global Executive Composite, MI: Metacognitive Index, and TBI: Traumatic Brain Injury.

**Table 9:** Associations between main indexes on BRIEF-A and symptom scales on ASR at 2–5 years after moderate and severe TBI.

ASR symptom scales	BRIEF-A		BRIEF-A		BRIEF-A	
	Global Executive Composite (GEC)		Behaviour Regulation Index (BRI)		Metacognitive Index (MI)	
	*r*	*p* value	*r*	*p* value	*r*	*p* value
Anxious/depressed	0.75	<0.001	0.70	<0.001	0.72	<0.001
Withdrawn	0.59	<0.001	0.55	<0.001	0.57	<0.001
Somatic complaints	0.51	<0.001	0.50	<0.001	0.47	<0.001
Thought problems	0.44	<0.001	0.43	<0.001	0.41	0.001
Attention problems	0.86	<0.001	0.77	<0.001	0.83	<0.001
Aggressive behaviour	0.62	<0.001	0.72	<0.001	0.49	<0.001
Rule-breaking behaviour	0.44	<0.001	0.40	0.001	0.43	<0.001
Intrusive behaviour	0.20	0.112	0.27	0.027	0.12	0.319

ASR: Adult Self-Report; BRIEF-A: Behaviour Rating Inventory of Executive Function-Adult version.

## Appendix

See Figures 1 and 2 and Tables 7, 8, and 9.
